# Health workers’ perspectives of hepatitis B-related stigma among Aboriginal and Torres Strait Islander people in New South Wales, Australia

**DOI:** 10.1186/s12954-023-00844-4

**Published:** 2023-08-26

**Authors:** Elena Cama, Mitch Beadman, Kim Beadman, Max Hopwood, Carla Treloar

**Affiliations:** Centre for Social Research in Health, UNSW Sydney, Sydney, NSW 2052 Australia

**Keywords:** Hepatitis B, Stigma, Aboriginal and Torres Strait Islander people

## Abstract

**Background:**

Experiences of stigma and discrimination can act as a significant barrier to testing, monitoring, and treatment for hepatitis B virus (HBV). Aboriginal and Torres Strait Islander Australians are a population disproportionately impacted by HBV and yet limited research has explored HBV-related stigma in these communities. To begin preliminary explorations of HBV-related stigma among Aboriginal and Torres Strait Islander people, we interviewed health workers about their perceptions regarding HBV infection and HBV-related stigma.

**Methods:**

Participants were recruited from staff involved in the Deadly Liver Mob (DLM) program which is a health promotion program that offers incentives for Aboriginal and Torres Strait Islander clients to be educated on viral hepatitis, recruit and educate peers, and receive screening and treatment for blood-borne viruses (BBVs) and sexually transmissible infections (STIs), and vaccination. Semi-structured interviews were conducted with 11 Aboriginal and Torres Strait Islander and non-Aboriginal or Torres Strait Islander health workers who have been involved in the development, implementation, and/or management of the DLM program within participating services in New South Wales, Australia.

**Results:**

Findings suggest that stigma is a barrier to accessing mainstream health care among Aboriginal and Torres Strait Islander clients, with stigma being complex and multi-layered. Aboriginal and Torres Strait Islander people contend with multiple and intersecting layers of stigma and discrimination in their lives, and thus HBV is just one dimension of those experiences. Health workers perceived that stigma is fuelled by multiple factors, including poor HBV health literacy within the health workforce broadly and among Aboriginal and Torres Strait Islander clients, shame about social practices associated with viral hepatitis, and fear of unknown transmission risks and health outcomes. The DLM program was viewed as helping to resist and reject stigma, improve health literacy among both health workers and clients, and build trust and confidence in mainstream health services.

**Conclusions:**

Health promotion programs have the potential to reduce stigma by acting as a ‘one stop shop’ for BBVs and STIs through one-on-one support, yarning, and promotion of the HBV vaccine, monitoring for chronic HBV, and treatment (where required).

## Introduction

Health-related stigma refers to a social process by which people devalue or exclude others on the basis of their perceived health condition or features associated with that health condition [1]. Hepatitis B virus (HBV) is a blood-borne virus, with two main transmission routes: vertical transmission occurring perinatally (mother-to-child at birth), and horizontal transmission occurring after birth through exposure to blood or sexual fluids where the virus is present [2]. In the context of HBV, stigma may be related to negative attitudes towards potential transmission routes (e.g., unprotected sex, shared use of equipment for injecting drugs) [3], as well as fears around the infectiousness of the virus [3, 4]. Smith-Palmer et al. [3] recently conducted a literature review on stigma among people living with HBV, and found social stigma was common as was the internalisation or acceptance of negative judgements towards the virus by people living with HBV (i.e., internalised stigma). Stigma can also be enacted in the form of discrimination, such as the denial of health care, workplace discrimination, and exclusion by family, friends, or intimate partners [3]. There is evidence to indicate that stigma is a barrier to screening for HBV [5, 6] and access to monitoring and treatment (where needed) [3, 7–10].

HBV affects approximately 257 million people or 3.5% of the world’s population [11]. It is a primary risk factor for liver cancer [12], and one of the leading causes of mortality related to cancer [13]. In Australia, an estimated 1% of the population or 233,947 people are living with chronic HBV (when an infection persists for 6 months or longer) [14]; however, it is estimated that only around 68% of people living with chronic HBV in Australia have been diagnosed [15]. Treatment for chronic HBV can reduce the risk of cancer by up to 75% [16] and is a cost-effective public health strategy in at-risk populations [17], although not all people will require treatment. Ongoing monitoring of HBV (every six months) is recommended within clinical guidelines; however, data suggest that only 11.9% of people living with chronic HBV were receiving the optimal annual monitoring [18]. Aboriginal and Torres Strait Islander people represent 3.2% of the Australian population [19], yet estimates suggest that 7% of the population of people living with chronic HBV are Aboriginal and Torres Strait Islander people [15, 20]. Rates of HBV overall are declining in Australia due to universal vaccination [21], but despite this HBV notifications were more than one and a half times higher among Aboriginal and Torres Strait Islander compared to non-Aboriginal or Torres Strait Islander people in Australia in 2020 [20], and some researchers suggest that transmissions may be occurring both vertically and horizontally among Aboriginal and Torres Strait Islander children [22].

The National Aboriginal and Torres Strait Islander BBV and STI and the National Hepatitis B strategies have a reported goal to eliminate the negative impacts of HBV and other BBV and STI-related stigma and discrimination on people’s health, and recognise that Aboriginal and Torres Strait Islander communities face multiple layers of stigma [23, 24]. However, there is limited research on HBV-related stigma generally, and particularly among Aboriginal and Torres Strait Islander communities [9]. Most of the evidence that exists on HBV and stigma relates to migrant communities. For instance, research suggests that migrant communities contend with a range of structural and systemic barriers that impede access to HBV testing, monitoring, and treatment. These may include racism, a lack of culturally and linguistically diverse resources, a lack of culturally competent care, and a health system that does not recognise differences in cultural beliefs about health care and treatment [3, 8, 25]. Additionally, health workers may have concerns about treating clients with HBV, including that they, as health workers, do not have the skills or knowledge to treat these clients, and that clients may not understand the need for ongoing monitoring and care [26].

Research has shown that structural and systematic barriers, such as prohibitive cost of care, inappropriate location of services, lack of transportation, and lack of culturally appropriate and sensitive care, impede access to primary care more broadly among Aboriginal and Torres Strait Islander people [27]. Although there is limited research in the context of HBV specifically, there is evidence to suggest, for example, that health workers who provide care to Aboriginal and Torres Strait Islander communities may not have the appropriate knowledge about HBV and available treatment [28]. There is very little research documenting HBV-related stigma among Aboriginal and Torres Strait Islander people. One study indicated that Aboriginal and Torres Strait Islander people describe HBV using negative terminology, suggesting that stigma surrounding HBV persists [29]. Taken together, these barriers can impede access to testing, monitoring, and treatment (where needed), and result in low health literacy of HBV among Aboriginal and Torres Strait Islander people and health workers, mistrust in mainstream health service providers, and may result in further stigmatisation around HBV.

Noting the above impediments to client participation in health care, the Deadly Liver Mob program (DLM) has set out to overcome these barriers for Aboriginal and Torres Strait Islander communities in New South Wales (NSW), Australia. DLM is an incentivised health promotion program offered in three sites in Sydney metropolitan area and six in rural and regional NSW. The program aims to increase access to testing and treatment for blood-borne viruses (BBVs) and sexually transmissible infections (STIs) among Aboriginal and Torres Strait Islander communities across the state. The program offers incentives for Aboriginal and Torres Strait Islander clients to be educated on viral hepatitis, recruit and educate peers, and receive screening and treatment for blood-borne viruses (BBVs) and sexually transmissible infections (STIs), and vaccination for hepatitis A and HBV. This program was developed and implemented in partnership with Aboriginal and Torres Strait Islander people, for Aboriginal and Torres Strait Islander people, as a culturally appropriate and sensitive and safe way of improving access to screening and treatment of BBVs and STIs. Findings from an early evaluation of two pilot sites running DLM found that attendance of Aboriginal and Torres Strait Islander clients at the two health services significantly increased following the introduction of the program, and acceptability of the program was high among both staff and clients [30]. One of the key strengths of the program cited by workers and clients alike is the involvement of Aboriginal and Torres Strait Islander workers, who deliver the DLM program in a culturally sensitive and non-judgemental environment, overcoming some of the barriers to health care that exist for Aboriginal and Torres Strait Islander people, including stigma and discrimination [30]. Thus, this program and the health workers who deliver it can provide unique insights into HBV and HBV stigma among Aboriginal and Torres Strait Islander people, including identifying barriers to screening and treatment, as well as identifying ways that mainstream health care could better engage with Aboriginal and Torres Strait Islander clients. The aim of this paper is therefore to explore Aboriginal and Torres Strait Islander and non-Aboriginal or Torres Strait Islander health workers’ perspectives on HBV and HBV-related stigma, using data drawn from an evaluation of the DLM program.

## Methods

This paper reports on qualitative data, drawn from a broader mixed-methods evaluation of the DLM program, conducted by the Centre for Social Research in Health at UNSW Sydney [31]. The broader evaluation aimed to evaluate the effectiveness of the DLM program in increasing access to BBV and STI testing among Aboriginal and Torres Strait Islander people, to examine client and provider attitudes and barriers towards acceptability of the program, and to develop a scale-up plan and implementation toolkit to guide future sites. While hepatitis C (HCV) is the core focus of the program, the DLM program seeks to improve access to testing, treatment, and vaccination for other BBVs and STIs, including HBV.

An email invitation was sent by the research team to the HIV and Related Programs (HARP) Managers within the Local Health District (LHD) in NSW where DLM sites were located (seven LHDs in total covering nine DLM sites). HARP Managers then forwarded the invitation to the relevant staff involved in the development, implementation, and/or management of the DLM program within the participating services. The invitation email contained the Participant Information Statement providing additional information about the research and a consent form, and prospective participants were instructed to contact a member of the research team if they were interested in taking part.

Semi-structured telephone interviews were conducted with five Aboriginal and Torres Strait Islander and six non-Aboriginal or Torres Strait Islander health workers and key informants from five LHDs (total *n* = 11). In keeping with Braun and Clarke’s [32] approach to sample size selection and the issue of data saturation, sample size was largely a pragmatic decision, based on the exploratory nature of the research, the timeframe, and that these interviews were a subset of other interviews conducted as part of the research, with these interviews focussing on HBV. The interviews were conducted by one Aboriginal and Torres Strait Islander (MB) and one non-Aboriginal or Torres Strait Islander (MH) member of the research team between August and October 2020. Interviews explored the perspectives of health workers on HBV infection among Aboriginal and Torres Strait Islander clients and in the context of the DLM program, HBV-related stigma, and its impacts on both clients and staff within the program, and the potential for the program to improve health literacy and address the stigma surrounding HBV. Interviews were between 20 and 40 min duration. All interviews were audio-recorded and transcribed, with any identifying information about the participant removed from the transcript. Ethics approval for the evaluation of the DLM program was obtained from South Eastern Sydney LHD and the Aboriginal Health and Medical Research Council Ethics Committees. Site-specific approvals were also obtained from each LHD involved in the study.

Thematic analysis of the transcripts was conducted by experienced qualitative researchers (MH, EC). In this paper, we are interested in individual and structural factors which impact on HBV stigma. This is important as stigma research has thus far largely focused on individual or interpersonal factors [33]. However, structural factors must be considered in exploration of health conditions of groups experiencing significant disadvantage and racism. We used health literacy as a means to explore workers’ perspectives of community understandings of HBV. However, like Christie [34] we take the position that ‘effective health literacy is largely to do with effective communication’ (p. 40). Thus, when we speak about clients’ HBV health literacy, it must be understood in the context of whether there is effective communication from health systems to communities, rather than focusing on an individual’s lack of knowledge about complex health problems.

We complemented our analytic framework with analysis of intersecting stigma. Intersecting stigma and discrimination acts as a significant barrier to accessing mainstream health care broadly, with racism and particularly institutional racism being one of the key concerns [50]. Institutional racism refers to the ways in which our health systems are both intentionally and unintentionally steeped in racist beliefs and values [35], including through policies and processes that serve to discriminate against racial minority groups and maintain and reproduce health inequalities. For First Nations people globally, institutional racism manifests in various ways in health systems, through cultural misunderstandings that undermine the quality of care provided, poorer health outcomes including shorter life expectancy of Aboriginal and Torres Strait Islander people compared to non-Aboriginal or Torres Strait Islander people, longer waiting times for medical care, and reduced likelihood of receiving health treatments [36–38].

Finally, as research should avoid furthering the discourse of ‘deficiency’ or ‘pathology’ of Aboriginal and Torres Strait Islander people and communities [39], our analysis focused on aspects of strength and resilience. Thus, we focus on the strategies that workers have used to resist and address HBV health literacy and stigma in their work.

Two authors (MH, EC) closely read each transcript to produce data summaries informed by conceptual tools outlined above guided by Braun and Clarke’s approach to thematic analysis [40]. These summaries explored issues within the data aligned with our approaches to health literacy and stigma (particularly structural and intersectional stigma). These data summaries were used in a number of capacity building workshops attended by Aboriginal and Torres Strait Islander authors (MB, KB) and non-Aboriginal or Torres Strait Islander authors (EC, CT) to review interpretation and presentation of the findings. All authors reviewed the findings. Due to the small sample size and the close-knit community of people involved in the DLM development, implementation, and management, demographic characteristics of the sample were not collected to protect the confidentiality of participants.

## Results and discussion

The sample consisted of five Aboriginal and Torres Strait Islander and six non-Aboriginal or Torres Strait Islander health workers and key informants involved in the DLM program. Health workers were involved in varying aspects of the DLM program, from the initial development, planning, implementation, and delivery (including frontline Aboriginal and Torres Strait Islander workers and clinical staff). This section presents the findings from interviews with the 11 health workers as well as our interpretation of how these findings relate to the broader evaluation of DLM as well as existing literature. We refer to ‘viral hepatitis’ more broadly due to the challenges in disentangling stigma related to HBV and HCV, as well as the intersecting nature of stigma.

### Intersectional stigma surrounding viral hepatitis

Among Aboriginal and Torres Strait Islander clients of the DLM program, stigma is complex and intersectional. Aboriginal and Torres Strait Islander people often contend with multiple layers of stigma in their lives [41], so HBV-related stigma, reportedly, did not stand out as a specific problem. Instead, it was described by health workers as just one dimension within a cascade of intersecting stigma and discrimination, particularly racism.

I mean there are so many forms of stigma that conflate into and comes down upon an individual’s head and on a community shoulders, that being able to unpick … Being treated shabbily by health care workers or community members or family members and peers, because of an individual kind of health concern … there’s so much intersection and potential to elicit a stigmatised reaction from a whole range of different concerns, I don’t reckon people can have a particular need to identify it coming from one particular virus. People just are treated like shit all over the place. (Non-Aboriginal or Torres Strait Islander health worker)

In a meta-narrative systematic review of Aboriginal and Torres Strait Islander peoples’ experiences of health care, Jones, Heslop, and Harrison [42] found that client experiences were in part informed by the level of trust that Aboriginal and Torres Strait Islander people have in health systems. Trust was informed by past and current experiences within health care settings, but also historical traumas, including past experiences with non-health-related institutions or through government policies that adversely impact Aboriginal and Torres Strait Islander peoples. The researchers also found that mistrust in health systems manifests through scepticism around health information, mistrust in the transfer of private health information between services, and reluctance to share information with their health providers [42]. Similarly, in this research, as a result of intersecting stigmas and institutional racism, Aboriginal and Torres Strait Islander clients were reported as often not trusting ‘the system’ or the doctors who work in it, with many having experienced stigma and discrimination from health care services, such as hospitals. It was reported by health workers in this study that doctors and other health workers represent authority, which is challenging for some Aboriginal and Torres Strait Islander clients to trust given the devastating impacts of colonisation in Australian history, including ongoing inequality and racism. Treloar et al. [41] elsewhere described Aboriginal and Torres Strait Islander people describing ‘automatic’ expectations that their communities would have stigmatised health conditions such as hepatitis C, with these expectations potentially imposed by health workers but which can become internalised as well. As one client in their research said, ‘I think I would have to say that the automatic racist attitude that, you know, you'll come up against, you know, like, I expect you to have these things, you are black’. (p. 24). Thus, institutional racism represents an ongoing structural and systemic barrier for Aboriginal and Torres Strait Islander communities to access primary health care.

Intersecting stigma also referred to the stigma that surrounds viral hepatitis, including both hepatitis B and C. This was perceived to be driven by shame about specific social practices, and by fear of unknown transmission risks and health outcomes. For instance, participants perceived the stigma to surround HBV due to the broader stigma that surrounds viral hepatitis more broadly. Each type of viral hepatitis was perceived to be stigmatised due to their association with one another.

Hep [Hepatitis] B, the stigma around hep B … I mean, it’s just the hep word. (Aboriginal and Torres Strait Islander health worker)

I know there is stigma, there’s stigma with any hepatitis for anybody and it’s real and I hate to say that people don’t … people especially even health care workers can have stigma. I’ve seen it firsthand or treat people differently with hepatitis whether it be B or C… (Non-Aboriginal or Torres Strait Islander health worker)

Such stigma was also linked to negative attitudes around practices that may place people at risk for viral hepatitis transmission, such as injecting drug use and sexual practices. HBV is most commonly transmitted perinatally or vertically (mother-to-child at birth) [2], with most historical transmissions of HBV in Aboriginal and Torres Strait Islander communities occurring perinatally or in early childhood [43]. Despite this, the association between HBV and HCV meant that the focus among Aboriginal and Torres Strait Islander people was on transmission through injecting drug use and sexual intercourse. Health workers reported that Aboriginal and Torres Strait Islander communities often feel shame about injecting drug use and sexual practices, and thus HBV stigma is fuelled by its association with these practices. The stigma that surrounds HCV is largely fuelled by negative attitudes towards injecting drug use [44, 45], and thus it was no surprise that participants spoke of the shame among Aboriginal and Torres Strait Islander communities that surrounds injecting drug use. Most of the clients who enter the DLM program do so because they have a history of injecting drugs or are otherwise at risk of HCV, and thus it is difficult to disentangle the stigma that surrounds HBV and HCV among this client group. As the second health worker highlights below, they were unsure of whether HBV was seen as worse than HCV due to potential transmission via sexual intercourse or injecting drugs.

I think you know it’s also a bit shame in some Aboriginal communities around injecting drug use, so in terms of transmission and how the transmission occurred, was it sexually transmitted or was it from sharing injecting equipment or you know any IV [intravenous] use, so I think, yeah, there is a lot of shame around that. (Non-Aboriginal or Torres Strait Islander health worker)

You know the fact that it’s sexually transmitted, rather than just injecting related, you know like to some people it might make it a worse disease or a more dirty disease to have, whereas to other people, it’s like ‘oh no, I didn’t get it from needles, I just had unprotected sex’ so I think there’s a lot of value judgement going on there. (Non-Aboriginal or Torres Strait Islander health worker)

### The connection between viral hepatitis health literacy and stigmatisation

Health workers perceived that there were significant knowledge gaps in relation to viral hepatitis, which begin with health workers and extend to the general community. This is supported by prior research, which reports low levels of HBV knowledge among both health workers and Aboriginal and Torres Strait Islander people in Australia [28, 46]. Workers discussed that there is a general lack of understanding of the differences between different types of viral hepatitis, the availability of a vaccine for HBV, and the varying treatment options. This is supported by previous research in a community sample which found that HCV knowledge may be better than HBV knowledge, with some important gaps existing in relation to vaccines and treatment [47]. Although DLM program staff were well informed about viral hepatitis in general, participants noted the complexities in understanding HBV and acknowledged that it had taken them quite some time to understand the differences between hepatitis A, B, and C.

Not a lot of them know about A, B, and C, I mean, it’s a big thing for even us to [understand] you know the differences between A, B, and C even as health workers trying to work out in the brain what’s the difference. (Aboriginal and Torres Strait Islander health worker)

I think that’s tricky [laughs]. I think that’s really tricky and I don’t know that that’s limited to Aboriginal community, because I think generally people get very confused and it’s true to say that even at the very beginning with our frontline project workers, we took a long time to really drill down with them what the differences were. (Non-Aboriginal or Torres Strait Islander health worker)

As we have noted, HBV health literacy must be understood within the context of the presence or absence of effective communication between health systems and community [34]. Health systems must provide effective, culturally appropriate, and culturally safe education and resources to Aboriginal and Torres Strait Islander communities in order to disentangle complex medical information. Thus, it is no surprise that as a result of the broader knowledge gaps around HBV among health workers, viral hepatitis health literacy was said to be poor among Aboriginal and Torres Strait Islander people upon entry to the DLM program. Participants perceived that the lack of HBV knowledge was linked to a focus on HCV within the broader health sector, resulting in HBV not being seen as a priority for Aboriginal and Torres Strait Islander clients:

I don’t think [HBV is] a major issue for [Aboriginal and Torres Strait Islander clients]. I don’t think it’s a priority. Most of the conversations and most of the health messaging that we provide, a lot of focus is on building clients health literacy around blood-borne viruses, with a really sharp focus on hepatitis C, because the majority of people that we work with have a history of injecting drug use and just the relative priority that is placed on hep C a whole range of programs, both clinical, needle and syringe, the community sector. (Non-Aboriginal or Torres Strait Islander health worker)

This supports prior academic literature which highlights significant knowledge gaps around protective behaviours, transmission, monitoring, and treatment of the infection [29, 46].

Although relatively few clients tested positive for HBV, many clients were found to require HBV vaccinations (which are offered as part of the DLM program) despite being in the age groups in which they were expected to have received vaccination as part of Australia’s national immunisation program. Therefore, the lack of knowledge around the different types of viral hepatitis was concerning for health workers, who perceived that clients were unaware how serious this preventable infection can be. As the following quote shows, clients conflated HBV and HCV, and thus there was confusion about there being no vaccine available for HCV.

I think there’s still an enormous amount of confusion between B and C and A and even clients we have treated for hepatitis C will come in and say ‘I had my hep C vaccination’ or something like that, they don’t quite understand. I don’t think any of the clients realise how serious hepatitis B can be, because they think it’s just like hep C sort of that, but it’s not quite, and I have been very surprised on doing blood tests on the Deadly Liver Mob clients how many of the people are not immune to hepatitis B when they are in the age group where they should be. (Non-Aboriginal or Torres Strait Islander health worker)

Participants reported that there was confusion among clients about the transmission routes for HBV infection. For instance, some clients associated HBV infection with injecting drug use, and thus HBV infection was not widely understood to be sexually transmissible:

… it’s like you know when people are in fear of sexually transmitted infections, hepatitis B isn’t the top one that springs to your mind is it? Chlamydia, gonorrhoea and syphilis or something, you know top three, you are not going to name hep B. (Non-Aboriginal or Torres Strait Islander health worker)

Some of the health workers perceived that there was little to no HBV-related stigma among clients, in part due to a lack of available information about the infection. This was also because some health workers perceived that HCV was more highly stigmatised due to its association with injecting drug use. A lack of awareness about HBV can be a double-edged sword, as on the one hand people might be less inclined to stigmatise a condition they know nothing about, yet on the other hand, low viral hepatitis health literacy can facilitate processes of stigmatisation by exacerbating misguided fears and confusion about risks of contagion. This is supported by previous research which has found that stigma surrounding HBV is linked to fears of the contagiousness of the infection [3, 4].

Hep B, I don't think there is much stigma around that, because I don't think a lot of people understand that it’s very different from Hep C. See hep C has that injecting drug users stigma and I really don't believe that hep B is being talked about enough in the community, so the understanding is different and there is a vaccination and it just didn’t seem even to the elders that hepatitis B was in anyway stigmatised as hep C was. (Aboriginal and Torres Strait Islander health worker)

… [The DLM clients] don’t know much about [HBV infection], so and yeah … there’s not much stigma, because there’s not much information about it going around. Maybe there is, but a lot of Aboriginal people aren’t getting it, so … (Aboriginal and Torres Strait Islander health worker)

However, others reported that HBV was stigmatised largely due to its association and conflation with HCV. This had important implications for clients, with workers describing that HBV-positive clients had concerns about disclosing their positive status to potential sexual partners, and often attended the DLM program by themselves without a support person. This was understood by one health worker as an indication of stigma and the liminal status of HBV infection within Aboriginal and Torres Strait Islander communities: it is barely perceived as distinct from HCV infection, and people living with HBV infection fear they will be similarly stigmatised.

They know that they’ve got hep B and they’re concerned about sexual partners and future and whether that will affect their relationships… That’s the concerns especially for the person that knows that you know if they’re going to have a relationship they then need to cross that bridge and how they’re going to feel about … how that person is going to treat them and whether the relationship will go further or not you know. (Non-Aboriginal or Torres Strait Islander health worker)

### Strategies to reduce viral hepatitis-related stigma

In addition to proposing effective communication as core to health literacy, Christie [48] also suggests that culturally appropriate and culturally safe approaches that work towards a shared understanding of issues are more effective than imposing Western biomedical understandings of health. A culturally appropriate or safe approach would be one that develops respectful relationships between health systems and Aboriginal and Torres Strait Islander people, and which ‘is mindful of language, worldview, existing knowledge and beliefs’ [29] (p. 2). As others have noted, employing Aboriginal and Torres Strait Islander staff, ensuring community ownership, and engaging with community are critical to improving health care access and accessibility among Aboriginal and Torres Strait Islander communities [27, 49]. When participants spoke about strategies to reduce viral hepatitis-related stigma, they often referred to DLM as uniquely able to counter stigma and bridge the gap between Aboriginal and Torres Strait Islander people and mainstream health services, particularly due to the presence of a frontline Aboriginal and Torres Strait Islander DLM worker. The presence of an Aboriginal and Torres Strait Islander worker has elsewhere been described as a core strength of the DLM program in facilitating connections between clients and mainstream sexual health services [30]. In this context, health workers described that Aboriginal and Torres Strait Islander health workers could deliver health promotion messages around HBV screening, vaccination, monitoring, and treatment (where required) in culturally appropriate and culturally safe ways. Research suggests that yarning style interactions are positively received by Aboriginal and Torres Strait Islander clients because they are informal and relaxed, allowing clients to communicate as they would within their communities [42]. Within DLM, having honest conversations with clients about viral hepatitis through yarning, coupled with resources from community organisations, reportedly reduced stigma through clearing up misinformation:

I think that peer Aboriginal workers can probably deliver the message better than non-Aboriginal health workers, potentially they have got more of a connection with the clients, they can maybe relate it back to country and their mob better than you know hearing it from a non-Aboriginal person who may not understand the cultural significance of a vaccination for somebody and be able to explain it in a way that makes them feel culturally safe about making that decision to vaccinate themselves. (Non-Aboriginal or Torres Strait Islander health worker)

One health worker described the importance of the Aboriginal and Torres Strait Islander worker in establishing relationships with Aboriginal and Torres Strait Islander clients, as well as fostering constructive and respectful relationships with non-Aboriginal or Torres Strait Islander health staff and addressing health literacy concerns. In this way, DLM can act to build trust between Aboriginal and Torres Strait Islander communities and mainstream health services. Trust is critical in improving Aboriginal and Torres Strait Islander people’s access to and experiences within health care [42]. Trust and rapport take time to build, and they were described in this research as preconditions for addressing stigma in whatever shape or form it takes. Aboriginal and Torres Strait Islander DLM workers needed to build rapport and trust with Aboriginal and Torres Strait Islander clients before the clients felt comfortable enough to attend the DLM program or before they would consider visiting a liver clinic accompanied by an Aboriginal and Torres Strait Islander peer worker. This is particularly because beyond the frontline Aboriginal and Torres Strait Islander DLM worker, much of the staff within Australian health systems are non-Aboriginal or Torres Strait Islander. As the quote below illustrates, the Aboriginal and Torres Strait Islander workers have to date assisted in facilitating clients’ access to mainstream services, by accompanying clients to various appointments at a nearby hospital:

You know some people don't like going up to the hospital, they come to us and ask us to take them up to the hospital. (Aboriginal and Torres Strait Islander health worker)

Participants believed that education was critical to reducing the stigma that surrounds viral hepatitis. As the following health workers described, it is important to have open conversations about HBV both to raise awareness about the infection and treatment options, but also to address stigma, similarly to mental health:

I think it’s talking about it … it’s like anything else, it’s like I suppose mental health or anything else if people don't want to talk about it, they think it’s going to upset somebody, but you know anything about health you should talk about it openly… I think once you gain their confidence to say, you know, let’s encourage people to talk about things if they have an issue, because two brains are better than one if you work together, at the end of the day, you will get something that satisfies everyone you know… the word gets around, like you know, if you are going to educate one person to educate somebody else and it will go around, might take a while but it will get there I think sooner or later. Might take a while but I think it’s talking and education around stuff. (Aboriginal and Torres Strait Islander health worker)

I just think going out into the community, having our Aboriginal peer worker and visiting places where Aboriginal people meet and talking about it is perhaps the best way and talking about what we do. (Non-Aboriginal or Torres Strait Islander health worker)

Because the core focus of the DLM program continues to be HCV, HBV infection generally remains a ‘hidden issue’ (Non-Aboriginal or Torres Strait Islander health worker). To date, efforts to reduce HBV-related stigma within the DLM program have mostly been opportunistic, such as when a client returns a positive test result and is given information to address their concerns around transmission to family and friends. Due to the intersecting nature of viral hepatitis stigma, health workers stressed the importance of educating clients on hepatitis A, B and C in future:

I know DLM is mainly about hepatitis C, but I believe that to understand hepatitis C, you need to educate on A and B as well so that they can be defined and separated from each other in the education. Yeah, that C is a very different strain I mean, when we started the project, there wasn’t a cure for hep C then, there wasn’t a vaccination and you know to let them know that there is a vaccination for hep A and for hepatitis B but if you got hepatitis B like chronically, there was no cure for that either. So I used to say to them, ‘you know, you are going to be screened and it will be hep B too and if they say that you need the vaccination, we will pay you to do that because once you have had the vaccination you are covered and you don’t want to get hep B because it’s lifelong’. (Aboriginal and Torres Strait Islander health worker)

It was suggested that HBV-related stigma might be addressed via a program to increase HBV testing, monitoring, and treatment (where required), and by focusing upon the prevention of liver cancer from uptake of the HBV vaccine. Given that stigma was believed to be fuelled by negative attitudes towards social practices that place people at risk of viral hepatitis, the following health worker suggested that focusing on the risk of cancer might help to alleviate viral hepatitis-related stigma:

It’s okay to talk about cancer but it’s not okay to talk about this virus in your blood. So, you know, that’s just a messaging thing, ‘Get screened and get tested and help prevent cancer’. And immunisation for hep B you know, ‘It’s an anticancer vaccination’ is basically what you are asking people to do. That’s pretty impressive if you talk about that, so that’s a messaging thing, whether that really changes people’s feeling of stigma, I don’t know … (Non-Aboriginal or Torres Strait Islander health worker)

Participants reported that the DLM program has improved the lives of many clients and that it is important to use this model to build rapport, to reduce stigma and to open discussions about difficult topics, so that DLM appointments become an opportunity for clients to have a conversation rather than solely a medical consultation. This approach has been useful for addressing HCV-related stigma within Aboriginal and Torres Strait Islander communities. A similar reduction in stigma might be expected if the profile of HBV infection was carefully and sensitively increased within the DLM program. Although HCV is the focus of the program, ultimately, the DLM program was described as being a culturally appropriate and safe mechanism through which to address viral hepatitis health literacy more broadly, improve access to testing and treatment, and address other health concerns:

…understanding that the health and social needs and community needs of Aboriginal people need to be addressed as a whole, so working with individuals and communities and identifying and fostering constructive useful, respectful and authentic ways of working with Aboriginal people and then the mechanism by which we get to prioritise or we get to raise, you know discussing health literacy concerns around people with intravenous drug use, people at risk of viral hepatitis, mapping out … identifying what they are going to want to do with the information that we are providing and then setting a really clear and you know easy way to engage with the health service and working through the various health needs that are identified… DLM has been such an awesome mechanism for us, … so while hep C is basically the entry point, we are really keen to use that mechanism, that way of working, because it’s authentic, it works and it provides people with incentives, you know it’s wholly focused around the crucial role of the relationship between the Aboriginal health worker and the Aboriginal client and supporting that and then enabling whatever comes through that assessment process to be managed effectively. (Non-Aboriginal or Torres Strait Islander health worker)

## Conclusions

Health workers in this research perceived that HBV was not a priority in Aboriginal and Torres Strait Islander communities, and this was partly due to the focus of health promotion programs on screening and treatment for HCV and the conflation among health workers and the community of the different types of viral hepatitis. Stigma is complex and may be intersectional or layered according to multiple identities or social practices [50]. Aboriginal and Torres Strait Islander communities face significant racism and discrimination more broadly [38, 51], and it can be difficult to pinpoint stigma that exists specifically in relation to viral hepatitis. Both racism and stigma surrounding HBV act as a significant barrier to Aboriginal and Torres Strait Islander communities accessing mainstream health services, thus further research is needed to attempt to disentangle the complex nature and impacts of viral hepatitis stigma within these communities.

The DLM program is uniquely situated in being able to reduce hepatitis-related stigma in Aboriginal and Torres Strait Islander communities through the one-on-one support provided to clients (including Aboriginal and Torres Strait Islander workers accompanying clients to appointments at hospitals and liver clinics), through yarning (including the education sessions delivered by Aboriginal and Torres Strait Islander health workers about viral hepatitis), establishing trust in mainstream health services (by Aboriginal and Torres Strait Islander staff introducing clients to any non-Aboriginal or Torres Strait Islander clinical staff), and by health workers engaging clients in HBV vaccination, assessment, monitoring, and treatment (where required). Elsewhere, we have demonstrated the success of the early pilot DLM programs in encouraging Aboriginal and Torres Strait Islander communities to go through the DLM ‘cascade of care’ (education, screening, results, additional treatment if required) [30]. Hla et al. [52] documented the positive impacts of a ‘one stop liver shop’ program (that includes Aboriginal and Torres Strait Islander health workers) in remote Aboriginal and Torres Strait Islander communities. Thus, the DLM model of care could be further developed to focus on HBV education and screening in Aboriginal and Torres Strait Islander communities, and thereby acting as a ‘one stop shop’ across all BBVs and STIs. While clients are screened for HBV and offered vaccination if applicable, the focus of DLM and the education provided to clients is currently on HCV. An expanded focus of the education component to include HBV might address the conflation between the different types of viral hepatitis that was noted in this study. Scholars have recently co-designed and evaluated culturally appropriate and safe HBV-related education for the Aboriginal and Torres Strait Islander health workforce, which could help guide such an expanded focus for the DLM program [53].

There are several study limitations of this research that should be noted. Firstly, the sample was recruited through the DLM program; a health promotion program that specifically aims to educate Aboriginal and Torres Strait Islander communities about BBVs and STIs and improve access to screening and treatment. The sample is not generalisable to all DLM workers within NSW, nor is it generalisable to health workers beyond the program. These are workers who have greater education and awareness of the various types of viral hepatitis, with interview participants noting that other health workers may lack such awareness and knowledge. Previous research has found that some health workers who provide care to Aboriginal and Torres Strait Islander communities lack knowledge about available treatment for HBV [28]. More generally, research has also found that health workers may be concerned that they lack the skills and confidence to treatment clients with HBV [26]. The DLM program is opportunistic in the sense that people are recruited to the program in relation to their potential risk of having HCV and are screened and treated for other BBVs and STIs in the process. Future research among health workers should diversify the sampling approach to include other health workers who may have contact with or treat clients living with HBV, and who may be unaware of the need to screen for the full suite of BBVs and STIs. This study was also limited in that we only interviewed Aboriginal and Torres Strait Islander and non-Aboriginal or Torres Strait Islander health workers about their perspectives of HBV and HBV-related stigma. These perspectives are valuable as a first step and to begin to map the field; however, future research should also seek perspectives of Aboriginal and Torres Strait Islander people to explore perceptions of HBV and HBV-related stigma within these communities. Finally, Aboriginal and Torres Strait Islander communities come from diverse language and cultural groups across Australia, and therefore may have different perceptions of HBV and stigma. Unfortunately, we were unable to explore such differences, and future research could seek to explore such variations across communities in Australia.

The data presented in this paper have important implications for health promotion interventions among Aboriginal and Torres Strait Islander communities. Although the DLM program more broadly aims to improve health literacy related to viral hepatitis among Aboriginal and Torres Strait Islander communities, it is apparent that the focus is on HCV. This results in confusion among both staff and clients about the different types of viral hepatitis. Given that the infrastructure is already in place, including the good will of health workers in wanting to improve knowledge and health care access in relation to HBV, it would be useful for the DLM to have a stronger focus on HBV education moving forward. Health promotion programs like DLM have the potential to reduce stigma by acting as a ‘one stop shop’ for BBVs and STIs, through employment of Aboriginal and Torres Strait Islander peers to increase trust in health services, one-on-one support, yarning, and promotion of screening, monitoring, treatment (where required), and vaccination.

## Data Availability

The datasets generated and/or analysed during the current study are not publicly available due to the possibility of individual privacy being compromised.

## Abbreviations

BBV: Blood-borne virus
DLM: Deadly Liver Mob
HARP: HIV and Related Programs
HBV: Hepatitis B
HCV: Hepatitis C
LHD: Local Health District
NSW: New South Wales
STI: Sexually transmissible infection
