# Correction: Health workers’ perspectives of hepatitis B-related stigma among Aboriginal and Torres Strait Islander people in New South Wales, Australia

**DOI:** 10.1186/s12954-024-00925-y

**Published:** 2024-01-13

**Authors:** Elena Cama, Mitch Beadman, Kim Beadman, Max Hopwood, Carla Treloar

**Affiliations:** Centre for Social Research in Health, UNSW Sydney, Sydney, NSW 2052 Australia

**Correction: Harm Reduction Journal (2023) 20:116** 10.1186/s12954-023-00844-4

Following publication of the original article [1], the reference 31 has been added to the reference list and the same has been shown below:

31. Treloar, C., Beadman, K., Beadman, M. et al. Evaluating a complex health promotion program to reduce hepatitis C among Aboriginal and Torres Strait Islander peoples in New South Wales, Australia: the Deadly Liver Mob. Harm Reduct J 20, 153 (2023). 10.1186/s12954-023-00885-9

The original article has been corrected.
